# Congenital medulloblastoma presented in the neonatal period

**DOI:** 10.4322/acr.2021.258

**Published:** 2021-04-26

**Authors:** Patricio García-Espinosa, Max Molina-Ayala, Edgar Botello-Hernández, Estefania Villareal-Garza, Álvaro Barbosa-Quintana

**Affiliations:** 1 Universidad Autónoma de Nuevo León, Hospital Universitario “Dr. José Eleuterio González”, Departamento de Neurología Monterrey, México; 2 Universidad Autónoma de Nuevo León, Hospital Universitario “Dr. José Eleuterio González”, Departamento de Patología y Citología, Monterrey, México; 3 Universidad Autónoma de Nuevo León, Facultad de Medicina, Hospital Universitario “Dr. José Eleuterio González”, Monterrey, México

**Keywords:** Brain Neoplasms, Medulloblastoma, Pathology, Radiology, Case Reports

## Abstract

Congenital medulloblastoma is a rare brain tumor that appears in less than 1% of pediatric patients. Congenital medulloblastoma has a poor prognosis and should be suspected in patients with clinical manifestations of hyporeactivity, slow suction reflexes, and the presence of hydrocephalus. Herein we present the case of a 12-day-old female newborn who developed non-communicative hydrocephalus, hyporeactivity, and hyporeflexia. Magnetic resonance imaging of her brain showed a heterogeneous and cystic mass on the posterior cranial fossa. A suboccipital craniotomy was performed. The histopathologic analysis reported a congenital medulloblastoma. She remained in hospital until her death at 112 days old. This is one of the first case reports with clinical-radiological and pathological documentation. Awareness of this diagnosis can allow prenatal intervention, rendering a better prognosis. This case report exemplifies the importance of good prenatal follow-up.

## INTRODUCTION

A congenital medulloblastoma is extremely rare compared with medulloblastomas in general. It is usually diagnosed postnatally, is a form of neoplasm that originates in the posterior fossa at the level of the cerebellum, and is a highly aggressive neoplasm characterized by undifferentiated cells.^1^

In general, congenital brain tumors—one of the rarest of all tumors reported in the literature (which includes medulloblastoma)—are usually diagnosed within the first 2 months of life.^2^ ^,^ ^3^ The incidence has increased over the past 20 years and accounts for 0.5-1.5% of all pediatric brain tumors; however, the level of incidence differs depending on the patient's country of residence. These tumors are different in histology from those reported in the first or second year of life; teratoma appears to be the most common, followed by astrocytomas or primitive neuroectodermal tumors as reported by Wakai et al.,^4^ Sugimoto et al.,^5^ and Alamo et al.^6^ Even in this large case series, medulloblastoma tumors are rare. The clinical presentation is insidious, and the condition tends to have a high mortality rate; however, in the future, the prognosis is expected to improve because of prenatal diagnosis due to ultrasonography and magnetic resonance imaging (MRI).

This report presents one of the very first cases to use pathological images and imaging studies. The importance of this case report is that congenital medulloblastoma shows a strange pathology, which usually has a high mortality rate; it also can have a similar clinical presentation to other congenital brain tumors and shows a very similar pattern on imaging.

## CASE REPORT

**Figure 1:**
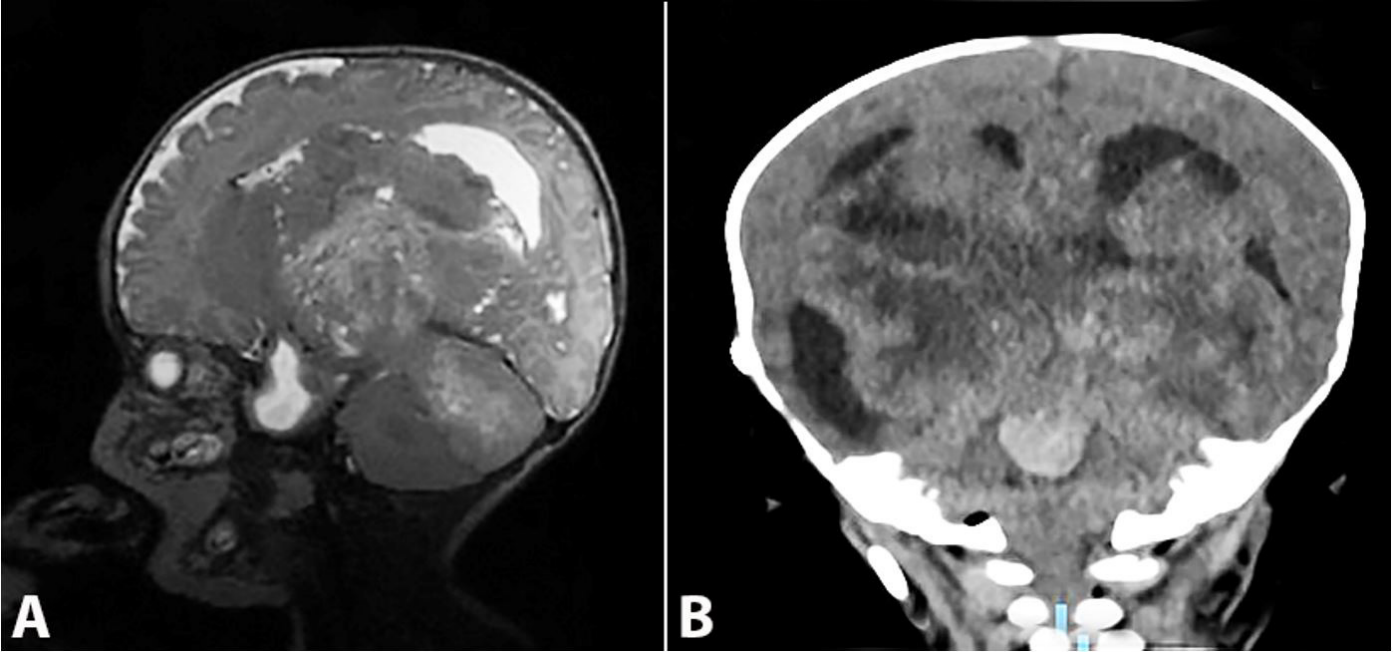
**A –** Magnetic resonance imaging, sagittal section: T2 sequence shows a heterogeneous cystic mass causing important dilatation of the ventricle system; **B –** Computed tomography of the coronal section, parenchymal (brain) window: shows a heterogeneous cystic mass causing important dilatation of the ventricle system.

A female baby was born by uneventful vaginal delivery at 36.5 weeks' gestation with an APGAR score of 8 and 9 at the first and fifth minutes, respectively. Her weight was 2.3 kg, her length was 48 cm, and her cephalic perimeter was 28 cm. The mother was 39 years old (gravida 6: three cesarean sections, two vaginal deliveries, one abortion) who attended prenatal consultation during the first trimester of pregnancy. On the baby's first day, she had ocular secretion, jaundice, hyporeactivity, and slow suction reflexes. On day 3, she presented six apnea episodes. A transcranial ultrasound reported obstructive hydrocephalus. The MRI (Figure 1A) showed a heterogeneous and cystic mass on the posterior cranial fossa, which compressed ventricles III and IV, causing severe dilatation of the ventricular system.

**Figure 2:**
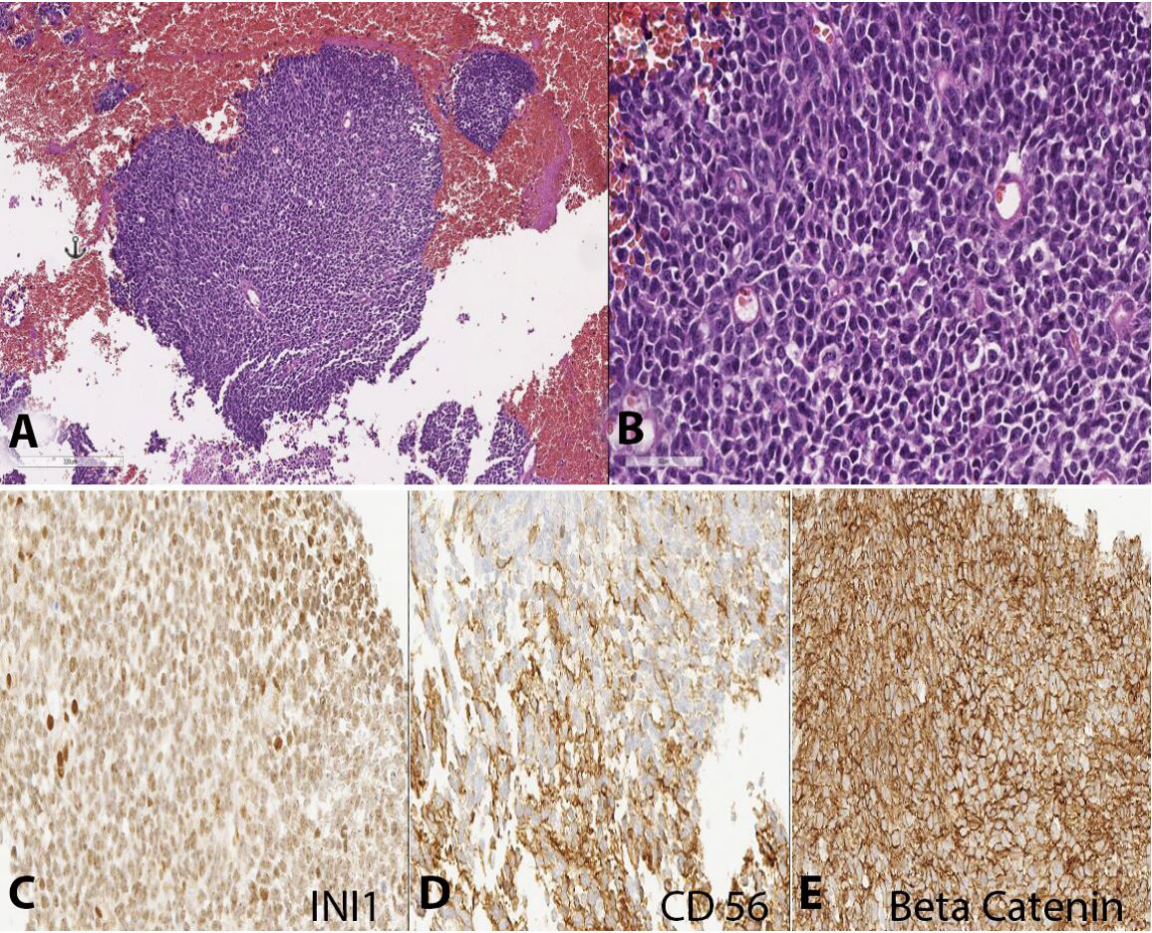
Photomicrographs of the brain tumor. **A –** Large sheets of small, round cells can be observed in the complete absence of any other components (H&E, 10X); **B –** High nuclear/cytoplasmic ratio, nuclear molding, and variability in the shape of nuclei are evident in this hypercellular neoplasm rendering a primitive, embryonal appearance (H&E, 40X); **C –** Retained INI1 (40X); **D –** Focal and partial reactivity for CD 56 in the neoplastic cells (40X); **E –** Beta-catenin membrane stain was positive (40X). c-MYC was negative. Additional stains for synaptophysin (focally positive), CD99 (negative), and KI67 (positive, 70%) were performed.

The patient was submitted to a suboccipital craniotomy, and the intraoperative pathological assessment depicted a primitive and undifferentiated neoplasm with neuroectodermal features. The final pathology diagnosis was medulloblastoma (WHO Grade IV; synaptophysin+, CD56+, beta-catenin+, and INI1+) (Figure 2).

The patient was submitted to an exchange blood transfusion on day 6, which resulted in clinical improvement. The neurosurgery team proceeded with a suboccipital craniotomy on day 21, resecting 40% of the total mass, which, on day 24, was complicated by a serous-draining fistula. One week later (day 31), the patient presented respiratory distress and oxygen desaturation due to *Klebsiella pneumoniae* pneumonia and required orotracheal intubation and an antibiotic regimen with vancomycin and levofloxacin. On day 33, she presented clinical improvement; however, 1 week later (day 40), the intracranial pressure increased again. Hyperchloremic metabolic acidosis ensued, and the fistula continued to drain serous-sanguineous fluid. The patient started with partial and focal seizures that manifested in the upper right limb and included sucking movements. Computed tomography of her brain was performed and revealed active bleeding and increased mass volume with medulla oblongata involvement (Figure 1B). Her clinical status worsened with desaturation, edema, hypothermia, and ultimately with mydriatic and areflexic pupils. Cardiopulmonary arrest and death were declared on day 112.

## DISCUSSION

Medulloblastoma, an embryonal neuroepithelial tumor of the cerebellum, is known to be the most common malignant brain tumor in Pediatrics (WHO Grade IV), representing 20% of all brain tumors in this age group. The outcome is poor, and a fatality occurs in one-third of all the patients with this condition despite the therapeutic improvements.^7^

The association of maternal diet and the higher risk of medulloblastoma was raised but not confirmed.^8^ ^,^ ^9^ However, viral infections and familial history of central nervous system tumors seem to be risk factors.^10^ ^-^ ^12^

Children are 10 times more likely to be affected by medulloblastoma than adults, and its incidence is calculated to be 1.5 per million population in the USA.^14^ Even so, congenital cerebellar medulloblastoma is extremely rare. The clinical presentation comprises respiratory distress, vomiting, hypoactivity, and high mortality.^13^ ^-^ ^17^

Symmetric ventriculomegaly and a midline cerebellar lesion (isointense with obstructive hydrocephalus) have been previously described and can be compared with our patient.^1^ ^,^ ^18^

Congenital anomalies and genetic syndromes have been described as being related to medulloblastoma; however, in a series of 173 patients with medulloblastoma, as reported by Evans et al.,^19^ no congenital medulloblastoma was reported.

In Hispanic literature, we found a few cases with similar clinical presentation to our case.^20^ ^,^ ^21^ The largest case series comprised just two cases of congenital medulloblastoma, where one patient was 5 months old.^22^ In a Spanish case series—in a Madrid hospital—where 9 neonatal tumors were reported from 1983 to 2001, only 1 was a congenital medulloblastoma.^23^ This is clearly because neonatal brain tumors, in general, are less than 1%.^24^ From 1948 to 1993 there were only 24 cases of congenital medulloblastoma, and all the patients except one died in the first 6 weeks.^25^

Other relevant diagnoses to take into account are teratoid/rhabdoid immature tumors (ATRT), a highly aggressive neoplasm that affects very young children, arises in the cerebellum and is composed of rhabdoid and other mesenchymal cells, and the loss of both copies of the INI1gene is it molecular signature; and embryological brain (PNETs), these tumors produce symptoms within the first 2 months of life (posterior fossa location) causing hydrocephalus, vomiting, and immunochemistry should be done to facilitate the tumor identification using the 2016 WHO classification of brain tumors.^26^ ^,^ ^27^ These immunochemistry stains included INI-1, synaptophysin, beta-catenin, among others, the molecular analysis also should be performed, genome-wide SNP microarray, and PCR should be done in autopsy and surgical CNS tumor specimen from all the infants under 6 months of age to determine the molecular characteristics that differ from tumors of older children, Viaene et al.^28^ recognizes the need to grouping medulloblastomas and atypical teratoid rhabdoid tumors separately from other embryonal tumors because the unique molecular profiles, in congenital tumors it is remarkable the presence of desmoplastic/nodular histological variant, but the classical histologic variant could also be seen, the most common location is the posterior fossa and the SHH activated TP53-wildtype is the most common molecular subgroup in congenital medulloblastoma (also is described to be the most common in infants), in classic medulloblastoma the most common molecular pathway is the one following MYCN amplification with isochromosome 17q and is presented in adults and older children as the one pathway following WNT-activated were beta-catenin is mutated and is related to Turcot Syndrome, classic medulloblastoma is considered a highly cellular tumor composed of diffuse masses of small, undifferentiated oval or round cells (as the one reported in our institution), the desmoplastic/nodular histological variant is permeated by fine collagen (reticulin) fibers with pale islands (center of the nodules that are reticulin-free) this histologic type is most common in infants and is considered to have a better prognosis (there is a direct relation between SHH-activated pathway and D/N histology and is related with Gorlin Syndrome)), it is important to remark that when appears, congenital medulloblastoma is within the first two months of life or prenatally, and some of them in the setting of a tumor predisposition syndrome such as Gorlin Syndrome, the study of Viaene et al.^28^ is one of the first in their type given principal importance to histologic and molecular characteristics of congenital tumors of CNS and illustrate the need of more studies of this specific characteristics to improve the future treatment and diagnosis and understanding.^29^ ^-^ ^31^

## CONCLUSION

Congenital medulloblastoma is one of the most uncommon malignant brain tumors that can be diagnosed before birth. In general, its clinical presentation, imaging, and the outcome are very similar. When this condition is suspected, an MRI should be performed before birth, which may permit intrauterine intervention, and thus render a better prognosis Molecular and genetic research should be done in central nervous system tumors typification to understand their unique characteristics and treatment.
